# Xpert MTB/RIF Ultra CT value provides a rapid measure of sputum bacillary burden and predicts smear status in patients with pulmonary tuberculosis

**DOI:** 10.1038/s41598-023-28869-6

**Published:** 2023-01-28

**Authors:** M. C. Martin-Higuera, G. Rivas, M. Rolo, I. Muñoz-Gallego, Paula Lopez-Roa

**Affiliations:** Department of Clinical Microbiology, Hospital Universitario 12 de Octubre, Madrid, Spain

**Keywords:** Microbiology, Diseases, Medical research

## Abstract

Traditionally, smear microscopy has been used to estimate bacillary burden in order to assess infectiousness in tuberculosis (TB) patients. Since Xpert MTB assays might replace smear microscopy as the first-line diagnostic test for pulmonary tuberculosis, an alternative measure of bacillary load that correlates with smear positivity is needed. This study assessed the correlation between C_T_ (with and without normalization), smear status, culture time-to-positivity (TTP), and clinical factors in patients with Xpert ultra positive sputum during a four-year period. A cut-off C_T_ value for smear positivity was also estimated. 204 samples were included. Strong correlation between both Xpert Ultra C_T_ values (raw and normalized) and smear status was obtained (r = 0.78 and − 0.79, respectively). The association between Raw-C_T_ and TTP was weaker than normalized-C_T_ (N-C_T_) and TTP (r = 0.50 and r = − 0.70, respectively). A Raw-C_T_ cut-off value of 21.4 was identified with 85.7% (95% CI 65.4–95) sensitivity and 92.9% (95% CI 84.3–96.9) specificity. A N-C_T_ cut-off value of 5.2 yielded a sensitivity of 94.3% (95% CI 86.2–97.8) and specificity of 85.7% (95% CI 65.4–95). Our study demonstrates that Xpert Ultra C_T_ value correlates well with other measures of bacillary load such as smear status or TTP. The correlation with TTP is stronger when the C_T_ value is normalized using the internal control. The proposed N-C_T_ cut-off value of 5.2 shows a better sensitivity than the Raw-CT when predicting smear positive status.

## Introduction

*Mycobacterium tuberculosis* (MTB) bacterial load in sputum plays an important role in determining disease severity and infectiousness in pulmonary tuberculosis^1–5^. Traditionally, smear microscopy for acid-fast bacilli (AFB) has been used to quantify mycobacterial burden at the time of diagnosis^6^. Time to positivity (TTP) measured in liquid culture provides an alternative way to quantify mycobacterial load, but requires longer turn-around-time^7^.

Considering the current recommendation of the WHO to replace sputum smear microscopy with Xpert MTB/RIF (Xpert) as an initial test in individuals suspected of TB, there is a need for an alternative measure of bacillary burden that has a good correlation with smear positivity. Xpert cycle threshold (C_T_) results provide a semi-quantitative measure of the bacillary load. The lower the obtained C_T_ values are, the higher the bacillary load is expected to be. Several studies have demonstrated a good correlation between C_T_ and more conventional measures of bacterial load, such as sputum smear grade and TTP in liquid culture^7–12^.

Xpert MTB/RIF Ultra assay (Xpert Ultra) is an upgraded version of the Xpert assay that incorporates two different multi-copy amplification targets for MTB complex detection (IS6110 and IS1081). Previous studies have described the analytic and clinical applications of the assay^13–15^, but few have evaluated Xpert Ultra as an indirect quantitative indicator for mycobacterial load^16^.

As the previous version, Xpert Ultra contains an internal control (IC) intended to detect suboptimal sample processing and PCR conditions that can modify the reproducibility between C_T_ values and bacterial load. As others (11), we hypothesize that a correction factor based on the IC could minimize experimental variability improving the correlation between C_T_ and other bacillary load measures.

The primary objective was to compare bacterial load quantitation by Xpert Ultra with smear grade and TTP, with and without incorporation of a correction factor based on IC. We also aimed to establish a cut-off C_T_ value to predict smear positivity.

## Material and methods

### Study design

We retrospectively reviewed all the sputum samples obtained between January 2018 and December 2021 from adult patients with clinical or radiological suspicion of pulmonary TB attending a tertiary care teaching hospital in Madrid, Spain.

All sputum samples received in the Microbiology Laboratory were tested with smear microscopy and mycobacterial culture with or without Xpert ultra. During the study period, Xpert Ultra was performed following negative smear microscopy results in patients with high clinical suspicion of pulmonary TB, and in patients who tested positive for smear microscopy in order to confirm the presence of MTB complex and to assess preliminary results for rifampicin resistance.

For the analysis, we only included patients with culture-confirmed pulmonary TB who had a positive result by Xpert Ultra and an available smear microscopy result. We reviewed data of those patients in which the three tests were performed simultaneously in the same sample. Patient’s data on Xpert Ultra test (semi-quantitative results and C_T_ values), mycobacterial culture, and smear microscopy were extracted from the laboratory information system. We also reviewed sociodemographic and clinical data including HIV status from medical records.

The Institutional Ethics Committee of Hospital Universitario 12 de Octubre approved the study. Due to the nature of the retrospective data review, and according with Regulation (EU) 2016/679 General Data Protection Regulation and the current legislation, the local committee waived the need for informed consent from individual patients. All study methods were carried out in accordance with the relevant guidelines and regulations established by the local committee.

### Bacteriological methods

#### Microscopy and culture

All sputum samples were digested-decontaminated following Kubica–Krasnow method^17^. Decontaminated samples were stained with auramine–thiazine red technique and examined with a fluorescence microscope by well-trained staff. Smears were graded according to the International Union Against Tuberculosis and Lung Disease (IUATLD) scale^18^.

Liquid cultures were performed by inoculation of 500 μL of decontaminated samples in mycobacteria growth indicator tubes (MGIT). MGIT tubes were incubated at 37 °C in an automated Bactec MGIT 960 instrument (Becton Dickinson, Sparks, MD, USA) for a maximum of 42 days. All positive cultures were confirmed to be MTB complex by detection of antigen MPT64 with a commercial immunochromatographic assay (BD MGIT™ TBc Identification Test, Becton Dickinson; Sparks, MD, USA). After identification of MTB complex strains, DST was performed using MGIT SIRE (Becton Dickinson; Sparks, MD, USA) according to the manufacturer's recommendations.

#### Xpert MTB/RIF ultra assay

The Xpert Ultra assay (Cepheid, Sunnyvale, CA) was performed adding sample reagent to the decontaminated specimen in a 2:1 dilution. 2.0 mL of the resulting mixture were then transferred to the Xpert Ultra cartridge and loaded into the standard four-module GeneXpert instrument. Results were interpreted by the GeneXpert software version 4.3. The semi-quantitative results of the Xpert Ultra assay were read as trace, very low, low, medium, or high. Trace result corresponded to samples that were MTB positive due to the presence of the IS6110 and/or IS1081 molecular signals in the absence of a signal from at least 3 of the *rpoB* probes.

As previously reported (13), we used the lowest C_T_ generated among the four *rpoB* probes during the nested-PCR stage of Ultra as a semi-quantitative measure of the MTB cell number in each test sample.

In order to be able to compare bacillary load between samples, we normalized Xpert Ultra C_T_ value (Raw-C_T_) with the IC-C_T._ This Normalized-C_T_ (N-C_T_) was calculated as the difference between IC-C_T_ value and Raw-C_T_ value (N-C_T_ = IC-C_T_ − Raw-C_T_).

Since tests that belong to the semi-quantitative “trace” category lacked *rpoB* detection, these results were excluded from the analysis involving C_T_ values.

### Statistical analysis

STATA V15.1 program was used for data analysis. Optimum cut-off value for smear positivity was calculated using a receiver operating characteristic (ROC) curve. Youden's index was calculated to establish the optimal C_T_ cut-off value to confirm/rule-in smear positivity. ROC curves were performed with Graph-Pad Prism V8.0 software.

Correlation between TTP, C_T_ values and smear positivity status was assessed using Spearman’s coefficient ρ with 95% confidence intervals.

Mann–Whitney test was conducted to assess whether the distribution of C_T_ values, TTP and smear positivity were related to clinical variables (fever, dyspnea, previous TB infection, cavitation, cough and hemoptysis).

### Ethical approval

The Institutional Ethics Committee of Hospital Universitario 12 de Octubre approved the study and due to the nature of the retrospective data review, waived the need for informed consent from individual patients.

## Results

During the study period, 674 patients underwent Xpert Ultra testing. Of those, 104 (15.4%) had a positive Xpert Ultra result and were included in the study. Sputum smear was positive in 69.2% (72/104) of the cases. The semi-quantitative results of Xpert Ultra were as follows: 48/104 (46.2%) high, 17/104 (16.3%) medium, 19/104 (18.3%) low, 7/104 (6.7%) very low and 13/104 (12.5%) trace.

Median age and Interquartile Range (IQR) of the patients with positive Xpert Ultra was 39 years (28–44) and 58.1% were men.

### Measures of bacillary burden

Smear negative samples had a median Raw-C_T_ of 25.5 (IQR 22–29) compared to 17.75 (IQR 17.3–18.8) for smear positive samples. Median N-C_T_ (IQR) for smear negative and smear positive samples were 1.8 (− 2.2 to 3.8) and 12.3 (9.4–13.9), respectively. Table 1 shows the distribution of Raw-C_T,_ N-C_T_ and IC-C_T_ according to each smear grade and Xpert Ultra semi-quantitative classification.

**Table 1:** Distribution of Raw-C_T,_ N-C_T_ and IC-C_T_ according to each smear grade and Xpert Ultra semiquantitative classification.

	C_T_ Value, mean (± SD)		
	Raw-C_T_	IC-C_T_	N-C_T_
**Smear grade**			
3 + (n = 20)	17.5 ± 0.1	31.5 ± 0.5	14.1 ± 0.5
2 + (n = 26)	18 ± 0.2	30.1 ± 0.5	12.3 ± 0.6
1 + (n = 22)	19.6 ± 0.5	28.1 ± 0.6	7.3 ± 1.8
Scanty (n = 3)	23. ± 2	26.7 ± 0.2	3.3 ± 1.7
Negative (n = 20)	25.4 ± 1	26.7 ± 0.5	1.7 ± 1.1
**Xpert ultra semiquantitative results**			
High (n = 48)	17.5 ± 0.4	30.9 ± 2.1	13.4 ± 2.2
Medium (n = 17)	19.1 ± 0.5	28.1 ± 1.8	9 ± 2.1
Low (n = 19)	23.4 ± 2.2	25.0 ± 6.4	1.5 ± 7.5
Very low (n = 7)	30.3 ± 2.3	22.5 ± 10	-7.7 ± 12.2

### Correlation of measures of bacillary burden

**Figure 1:**
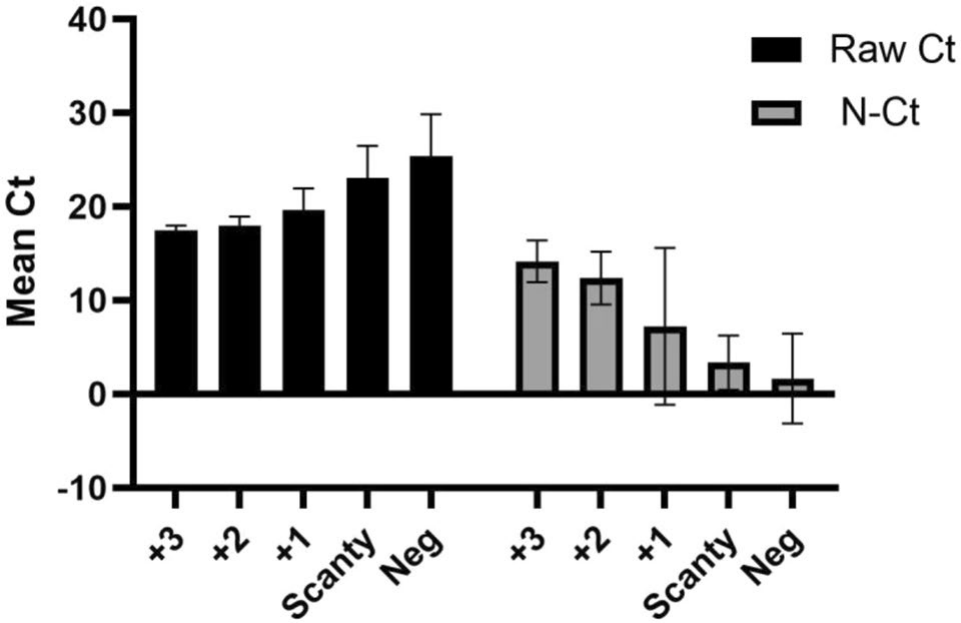
Mean (SD) cycle threshold (CT) distribution according to smear grade.

Spearman test for Smear positivity grade and C_T_ values was r = 0.78 (p < 0.005) for Raw-C_T_ and r = − 0.79 (p < 0.005) for N-C_T_. The relationship between each smear grade and the semi-quantitative results reported by Xpert Ultra assay is shown in Table 2. Figure 1 demonstrates the variation of mean Raw-C_T_, N-C_T_ and IC-C_T_ according to smear grade.

**Table 2:** Distribution of semi-quantitative Xpert Ultra results within each smear grade.

Xpert ultra result	Smear grade					Total
	3+, (%)	2+, (%)	1+, (%)	Scanty, (%)	Negative, (%)	
High, n (%)	20 (41.7)	19 (39.6)	8 (16.7)	0	1 (2)	48 (100)
Medium, n (%)	0	7 (41.2)	8 (47)	0	2 (11.8)	17 (100)
Low, n (%)	0	0	6 (31.6)	3 (15.8)	10 (52.6)	19 (100)
Very low, n (%)	0	0	0	0	7 (100)	7 (100)
Trace, n (%)	1 (7.7)	0	0	0	12 (92.3)	13 (100)
Total	21 (20.2)	26 (25)	22 (21.1)	3 (2.9)	32 (30.8)	104 (100)

**Figure 2:**
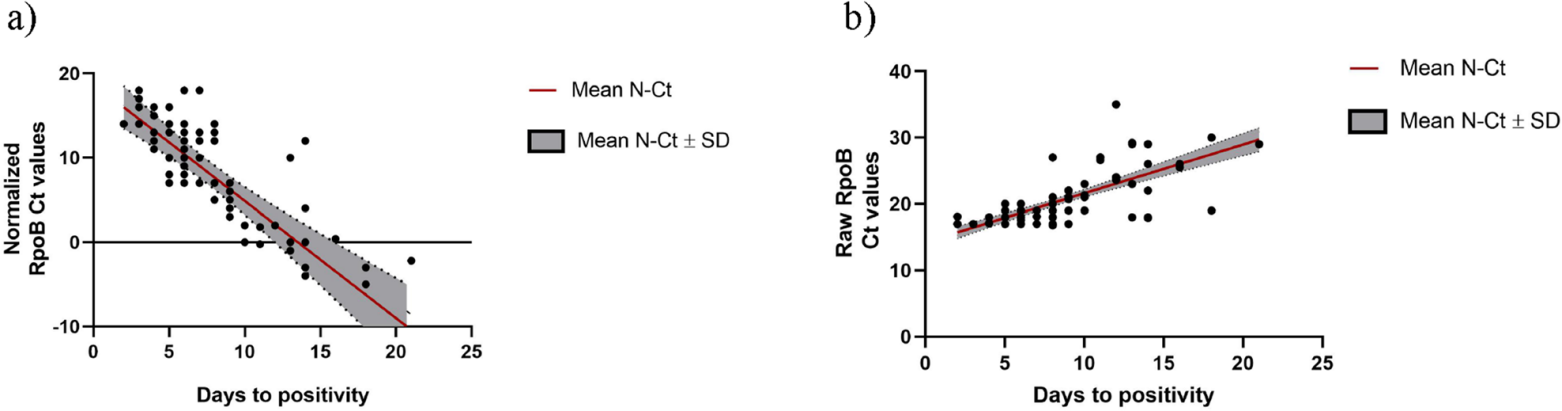
Spearman’s correlation study between (**a**) Normalized-C_T_ and TTP in liquid cultures. Spearman r coefficient = − 0.70, p value < 0.001; ( **b**) raw-C_T_ and TTP in liquid cultures. Spearman r coefficient = 0.52, p value < 0.001.

We also found a relatively strong correlation between both C_T_ values and TTP in liquid culture. The correlation was stronger for N-C_T_ (Fig. 2).

### CT cut-off values for smear positivity

Out of the Raw-C_T_ ROC curve analysis, an optimal cut-off value of 21.4 was identified with 85.7% (95% CI 65.4–95) sensitivity and 92.9% (95% CI 84.3–96.9) specificity. A N-C_T_ cut-off value of 5.2 yielded a sensitivity of 94.3% (95% CI 86.2–97.8), specificity of 85.7% (95% CI 65.4–95).

**Figure 3:**
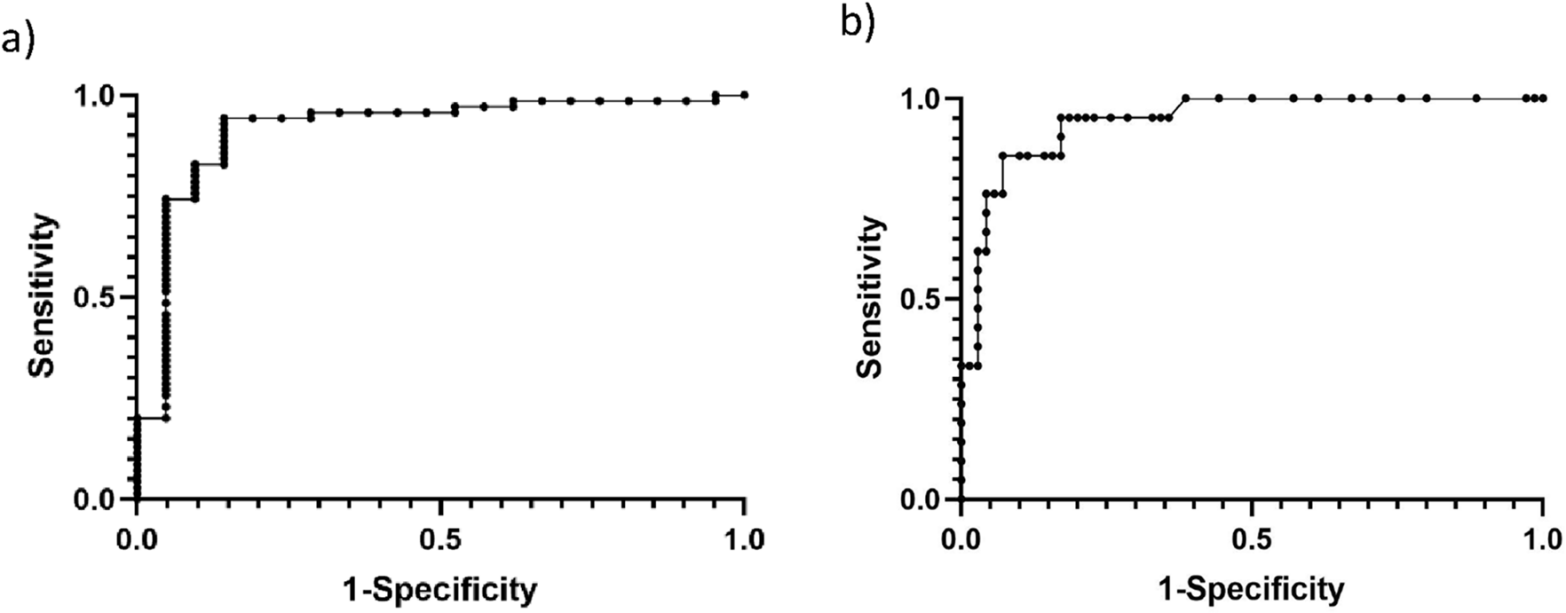
ROC curves analysis showing (**a**) N-C_T_, AUC = 0.91 (95% CI 0.84–0.93) and (**b**) Raw-C_T_ AUC = 0.94 (95% CI 0.88–0.98) values as a test for smear positivity.

Figure 3 shows the ROC curves of N-C_T_ and Raw-C_T_ threshold for smear positivity (AUC 0.91 and 0.94, respectively).

### Clinical variables affecting bacillary burden

A prior history of TB treatment was found in 13 (12.5%) patients. In all cases, the preceding treatment had been completed more than two years ago. Previous history of TB did not affect the relationship observed between C_T_ and bacillary load.

Cavitation was associated with higher bacillary burden as measured by all C_T_, TTP, and smear (Table 3).

**Table 3:** Univariate analysis (Mann–Whitney test) of clinical variables associated with bacillary burden according to mean C_T_ of Xpert Ultra, TTP and smear microscopy. Significant values are in bold.

Status (n)	Raw-C_T_		N-C_T_		TTP		Smear positivity	
	Median [IQR]	p value	Median [IQR]	p value	Median [IQR]	p value	N (%)	p value
Fever								
No (57)	18.9 [17.5–21.9]	0.16	9.6 [3.6–12.9]	0.24	8 [6–12]	0.20	37 (64.9)	0.42
Yes (47)	17.9 [17.3–19]		11.85 [6.6–13.4]		6 [5–9]		34 (72.3)	
Dyspnea								
No (78)	18.3 [17.5–21.4]	0.56	10.3 [4.6–13.6]	0.69	6.5 [5–12]	0.70	51 (65.4)	0.28
Yes (26)	17.8 [17.3–21.3]		12.4 [6–13.3]		6.5 [5.5–8.5]		20 (76.9)	
Previous TB infection								
No (91)	17.9 [17.3–20.6]	**0.02**	11.8 [6.5–13.6]	**0.02**	6 [5–11]	0.55	64 (70.3)	0.23
Yes (13)	20.6 [19–22.6]		5.1 [3–9.4]		9 [5.5–9.5]		7 (53.9)	
Cavitation								
No (40)	19.9 [17.8–23.6]	**0.03**	7.2 [2.5–12.3]	**0.015**	9 [6–13]	**0.001**	22 (55)	**0.022**
Yes (64)	17.8 [17.3–19.25]		12.1 [7.85–13.8]		6 [5–8]		49 (76.6)	
Cough								
No (20)	20 [17.1–23.2]	0.79	8 [2.5–13.7]	0.60	12.5 [5.5–15]	0.056	8 (40)	**0.0026**
Yes (84)	18.1 [17.5–20.6]		11.3 [6.5–13.3]		6 [5–9]		63 (75)	
Hemoptysis								
No (71)	17.9 [17.4–21.4]	0.49	11.9 [3.8–13.7]	0.58	6.5 [5–12.5]	0.60	46 (64.8)	0.26
Yes (33)	18.8 [17.5–21.35]		9.9 [5.65–13.05]		6.5 [5–9]		25 (75.8)	

*IQR* interquartile range. *TTP* time to positivity.

## Discussion

The present study demonstrates that Xpert Ultra C_T_ correlates well with smear grade and TTP in liquid culture and importantly, this correlation is stronger when a correction factor based on the IC is incorporated to minimize experimental variability.

Despite its suboptimal sensitivity, sputum smear microscopy remains the primary diagnostic tool for TB in most low-resource settings. In addition, grades of smear positivity are used to estimate mycobacterial burden to evaluate the infectiousness of patients in the context of screening and public health contact tracing^6^. Our results demonstrate that both Raw-C_T_ and N-C_T_ show a good correlation with smear microscopy grades (0.78 and − 0.79, respectively). As expected, despite the strong correlation between C_T_ and sputum smear grade, we found that Xpert Ultra semi-quantitative categories do not consistently differentiate sputum specimens into the same categories as smear microscopy grading does. This variability may be due to variations in smear classification or in CT reproducibility. Smear grading is operator-dependent and its sensitivity relies on variable operational issues. Therefore, and taking into account the normalization of C_T_ values based on IC, we suggest that Xpert Ultra could provide more robust results than smear grading.

Consistent with our previous study and others^6,11,12,19^, we also noticed that a significant number of sputum samples classified as smear-negative had relatively high mycobacterial load according to semi-quantitative result of Xpert Ultra. This finding suggests that a low C_T_ value could identify specimens with a high bacillary load, despite having a negative smear result. Since smear-negative patients are a substantial possible source of TB transmission^20,21^, Xpert Ultra quantitation might be used to identify the subset of potentially infectious smear-negative patients.

TTP is a better indicator than smear status respecting transmissibility and treatment response. In contrast, it is time consuming and expensive. We found relatively strong correlation between Xpert Ultra C_T_ value and TTP, particularly in the case of N-C_T_. However, consistent with previous studies^6,22^, we found that C_T_ value had a better correlation with smear grade, probably due to the detection of both viable and non-viable organisms compared to culture which only detects viable ones. This hypothesis is supported by data from previous studies^23,24^ strengthening the possibility that patients with previously treated TB are more likely to have positive Xpert results and negative cultures.

In order to use the C_T_ value as a surrogate measure for smear status, previous studies^10,19,25,26^ have proposed different C_T_ cut-offs to distinguish between smear-positive and smear-negative samples, obtaining variable sensitivity and specificity values depending on the study. In a recent meta-analysis, Xpert MTB/RIF C_T_ cut-off values of 31.8 and 27.7 were identified as predictors of smear positivity^25^. In our study, we identified two different cut-off values, one for the Raw-C_T_ (21.4) and the other for the N-C_T_ (5.2). Although both cut-off values accurately distinguish between smear-positive and smear-negative samples, the N-C_T_ cut-off value showed higher sensitivity when predicting positive smear status (94.3% vs. 85.7%). These findings indicate that Xpert Ultra N-C_T_ value has good clinical utility as a rule-in test for smear positivity. Due to the relatively high specificity, when used as a rule-out test, Xpert Ultra is likely to become clinically useful. Regarding the specificity value obtained, 14% of individuals above the proposed cut-off value would be smear-positive cases misclassified as smear-negative. These misclassified smear-positives patients are those with the lowest mycobacterial load and therefore, are less likely to transmit disease.

As for the impact of different clinical factors on bacillary burden, we observed that the only clinical variables associated with higher bacillary burden were cough and cavitation. These findings are likely related with higher sputum bacillary load present in patients with severe pulmonary disease^6^. We also found that history of previous TB infection was associated with higher C_T_ values. These associations between microbiological and clinical variables should be interpreted with caution because this approach did not consider confounding factors.

There are several limitations in our study. First, it was a retrospective non-multicenter cohort study, with a small number of smear positive samples. Second, since we only included patients with positive Xpert ultra we were not able to evaluate the TTP for those with Xpert negative results, however these patients are likely to have lower bacillary load and therefore a lower transmission risk. The cut off value of 5.2 should be evaluated in an independent cohort to demonstrate validity, and larger number of samples are needed to predict the cut-off value more accurately, especially in samples categorized as scanty gradation.

In summary, our preliminary findings, suggest that Xpert Ultra C_T_ value, adjusted to IC, could emerge as an alternative measure to estimate MTB load instead of smear status, with the recommended cut-off value of 5.2 to rule-in smear positivity. Laboratories might consider routinely reporting these values, although further studies should evaluate whether Xpert C_T_ values could predict infectiousness and transmission.

## Data Availability

The datasets generated during and/or analysed during the current study are not publicly available but are available from the corresponding author on reasonable request.
